# Insights from a 31‐year study demonstrate an inverse correlation between recreational activities and red deer fecundity, with bodyweight as a mediator

**DOI:** 10.1002/ece3.11257

**Published:** 2024-04-22

**Authors:** Martijn J. A. Weterings, Estella Y. C. Ebbinge, Beau N. Strijker, Gerrit‐Jan Spek, Henry J. Kuipers

**Affiliations:** ^1^ Van Hall Larenstein University of Applied Sciences Leeuwarden The Netherlands; ^2^ Wildlife Ecology and Conservation Group Wageningen University Wageningen The Netherlands; ^3^ Vereniging Wildbeheer Veluwe/FBE Gelderland/Natuurlijk Fauna Advies Mts Vaassen The Netherlands

**Keywords:** fitness consequences, human activity, risk effects, stress response, ungulates

## Abstract

Human activity is omnipresent in our landscapes. Animals can perceive risk from humans similar to predation risk, which could affect their fitness. We assessed the influence of the relative intensity of recreational activities on the bodyweight and pregnancy rates of red deer (*Cervus elaphus*) between 1985 and 2015. We hypothesized that stress, as a result of recreational activities, affects the pregnancy rates of red deer directly and indirectly via a reduction in bodyweight. Furthermore, we expected non‐motorized recreational activities to have a larger negative effect on both bodyweight and fecundity, compared to motorized recreational activities. The intensity of recreational activities was recorded through visual observations. We obtained pregnancy data from female red deer that were shot during the regular hunting season. Additionally, age and bodyweight were determined through a post‐mortem examination. We used two Generalized‐Linear‐Mixed Models (GLMM) to test the effect of different types of recreation on (1) pregnancy rates and (2) bodyweight of red deer. Recreation had a direct negative correlation with the fecundity of red deer, with bodyweight, as a mediator as expected. Besides, we found a negative effect of non‐motorized recreation on fecundity and bodyweight and no significant effect of motorized recreation. Our results support the concept of humans as an important stressor affecting wild animal populations at a population level and plead to regulate recreational activities in protected areas that are sensitive. The fear humans induce in large‐bodied herbivores and its consequences for fitness may have strong implications for animal populations.

## Graphical abstract

Recreation had a direct negative correlation with the fecundity of red deer, with bodyweight as a mediator. Our results support the concept that humans affect wild animal populations at a population level and plead to regulate recreational activities in protected areas that are sensitive.

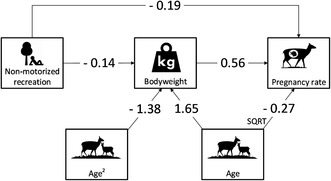

## 1 INTRODUCTION

During the last century, outdoor recreation (including off‐road activities) has strongly increased (Cordell, 2008; Larson et al., 2016; Stankowich, 2008). Between 2000 and 2012, recreation in protected areas globally increased by about 26% (Vallecillo et al., 2019). For example, in the Veluwe protected area in the Netherlands, the number of visitors increased 19‐fold between 1968 and 2005 and even increased 26‐fold in restricted areas (Bijlsma, 2006). Besides an increase in numbers, the types of recreational activities and their relative fraction have changed over time (Cordell, 2008). Ultimately, the intensification of recreational activities increased the encounter rate between recreationists and wild animals (Stankowich, 2008). Hence, this has become a growing matter of concern for wildlife conservationists and managers (Smith, Gaynor, & Suraci, 2021), which is also reflected in the increase in the number of studies (on average, 23.5% per year) that investigated the effect of recreation on wildlife between 1981 and 2015 (Larson et al., 2016; see also Marion et al., 2020).

Animals can perceive risk from recreational activities of humans similar to predation risk (i.e., ‘human‐caused predation risk’ hypothesis; sensu Frid & Dill, 2002; Smith, Gaynor, & Suraci, 2021). More specifically, fear of humans could even exceed the fear of large carnivores (Crawford et al., 2022; Darimont et al., 2015; Widén et al., 2022; Zbyryt et al., 2018). Antipredator responses due to recreational activities have been reported in several species, such as golden plover (*Pluvialis apricaria*) (Finney et al., 2005), puma (Puma concolor) (Smith, Suraci, et al., 2021), red deer (*Cervus elaphus*) (Mols et al., 2021), spruce grouse (*Falcipennis canadensis*), snowshoe hare (*Lepus americanus*), American marten (*Martes americana*) (Naidoo & Burton, 2020) and Indo‐Pacific bottlenose dolphin (*Tursiops aduncus*) (Bejder et al., 2006). Furthermore, certain types of recreational activities affect the risk perception of wild animals more significantly than others (Stankowich, 2008), such as hiking in comparison to driving a vehicle (Ciuti et al., 2012; Papouchis et al., 2001) and dog walking (Banks & Bryant, 2007). In general, animals perceive a higher risk from non‐motorized recreational activities compared to motorized activities (McLeod et al., 2013). For example, elk (*C. elaphus*) and pronghorn (*Antilocapra americana*) were less responsive in their behaviour to increasing levels of traffic in contrast to increasing levels of pedestrian activity (Brown et al., 2012).

Most studies have investigated changes in animal behaviour and distribution as a response to recreation (see, e.g. Berger, 2007; Visscher et al., 2023), whilst effects on species survival, fecundity or community ecology are less studied (Chitwood et al., 2022; Marion et al., 2020). Additionally, studies show contrasting effects of recreation on animals, which are probably related to differences in their duration (Marion et al., 2020). Overall, there are more ‘short‐term’ studies published (<5 years) (e.g., see: Jayakody et al., 2008; Marion et al., 2020; Naidoo & Burton, 2020) compared to ‘long‐term’ studies (>10 years) (e.g., see Nellemann et al., 2010; Yalden, 1990). Notwithstanding, long‐term studies generate important knowledge for management and conservation purposes (Naidoo & Burton, 2020; Pemberton et al., 2022), especially studies that get insight into the effects of recreation on species fitness. For example, during a 25‐year period (1980–2004), the survival probability of Allen Cays Rock Iguanas (*Cyclura cychlura inornata*) was negatively affected by tourist visitation (Iverson et al., 2006). Furthermore, the reproductive success of pine snakes (*Pituophis melanoleucus*) from 1986 to 2005 was lower in sites disturbed by off‐road vehicles compared to sites that were not disturbed (Burger et al., 2007).

**FIGURE 1:**
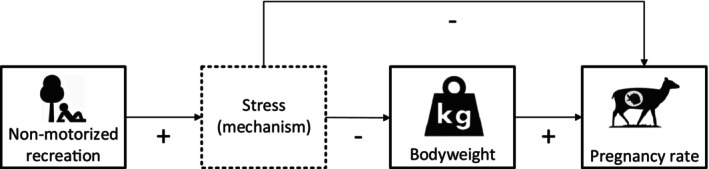
Conceptual representation of the effect of recreation on bodyweight and fecundity. Dotted box represents stress mechanism that can affect animal fecundity via two pathways.

Antipredator responses are diverse in the face of life histories, environmental conditions and individual variation (Caro, 2005; Cooper & Blumstein, 2018). Similar to predators (Weterings, Losekoot, et al., 2022), the presence of humans could affect the behaviour, stress response, energy expenditure and physiology of species, which in turn can influence the trade‐off between survival and fecundity (Creel, 2018; Frid & Dill, 2002). If unpredictable (e.g., in space or time), frequent encounters with humans could result in chronic stress (Boonstra, 2013; Creel, 2018) that could lead to increased energy expenditure, reduced food intake (Zanette et al., 2013), and negatively impact animal physiology and fecundity (Stankowich, 2008; Zanette et al., 2014). Apart from chronic stress, nutritional and energetic costs as a result of unpredictable encounters could affect fecundity, however, they are expected to be relatively weak and were not considered during this study (Creel, 2018). Nevertheless, chronic stress could reduce fecundity via two pathways (Zanette et al., 2011) (Figure 1). First, it could inhibit fecundity through a stress response that increases glucocorticoid levels (i.e., direct effect) (Bötsch et al., 2020; Whirledge & Cidlowski, 2010; Zbyryt et al., 2018). Second, it could affect bodyweight (Larson et al., 2016) and sequentially reduce fecundity (i.e., indirect effect) (Borowik et al., 2016). However, it is still unclear what the relative effects are of both pathways on fecundity are.

Therefore, the objective of our study was to gain insight into the relationships between recreation intensity and fecundity directly and between recreation intensity and fecundity indirectly via bodyweight. First, we hypothesized that fecundity is directly negatively related to recreation intensity (Bötsch et al., 2020; Whirledge & Cidlowski, 2010). Second, we hypothesized that stress, as a result of recreational activities, reduces bodyweight (MacLeod et al., 2014; Zanette et al., 2014) and thereby indirectly negatively affects fecundity (e.g., see: Borowik et al., 2016; Creel, 2018; Creel et al., 2014). Third, we expected that non‐motorized recreation has a larger negative effect on bodyweight and fecundity than motorized recreation (Brown et al., 2012; McLeod et al., 2013).

Our paper centres on the study of red deer (*C. elaphus*) that are relatively abundant in human‐dominated landscapes in Europe. As a large‐bodied ungulate, red deer may be specifically sensitive to human disturbance (Stankowich, 2008; Yalden, 1990). Red deer have been largely restricted to nature reserves in the central Netherlands that experienced an increase in the number of recreationists (see Bijlsma, 2006). During this study, we will focus on non‐motorized recreation, such as hikers, horse riders, dogs and cyclists, and motorized recreation, such as cars and quads. Except for the effects of predation risk on the fecundity of large deer (e.g., elk: Creel et al., 2007, 2009, 2011; white‐tailed deer (*Odocoileus virginianus*): Cherry et al., 2016) few studies investigate the effects of recreational activities on the fecundity of red deer (but see Putman & Langbein, 2003). However, quite a few studies focus on red deer behavioural responses to perceived risks of recreational activities (e.g., Marion et al., 2021; Mols et al., 2021).

## 2 MATERIALS AND METHODS

### 2.1 Study site

**FIGURE 2:**
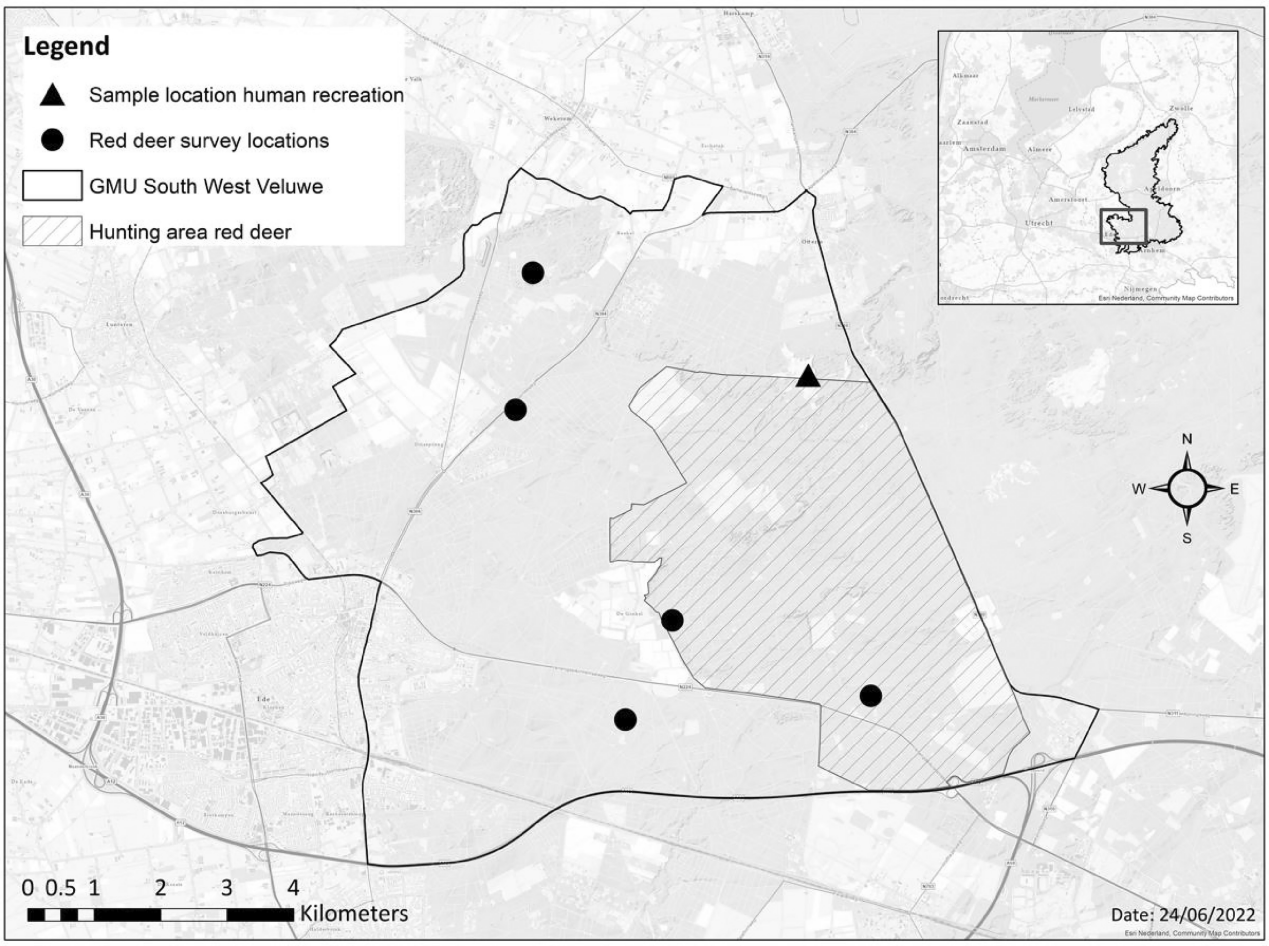
Overview of the study area with sampling locations (see data collection and preparation). Delineated in black: boundaries of the area of the Game Management Unit (GMU) (79.88 km^2^). Circles: monitoring locations of red deer. Triangle: sample location of recreational activities. Hatched surface: culling area of red deer. Inset: boundaries of the Veluwe including the study area. Data from Province of Gelderland (2021) and the Fauna Registration System (2022).

The Veluwe area (1250 km^2^) is designated as a protected area (i.e., Natura 2000) in the Netherlands (52°5′ N, 5°48′ E) (Figure 2). It has a temperate maritime climate, and its geology mainly consists of ice‐pushed ridges, fluvioglacial deposits and wind‐blown sands. The area is elevated 50–100 m above sea level, holding a large freshwater aquifer. Dry coniferous forest (i.e., mainly Scots pine [*Pinus silvestris*]), deciduous forest (i.e., mainly oak [*Quercus* sp.] and beech [*Fagus* sp.]) make up the majority of the habitat, interspersed with dry to moist heathland containing common heather (*Calluna vulgaris*) and grasses (Ten Houte & de Lange, 1977). Apart from red deer, three other ungulate species occur on the Veluwe, namely: wild boar (*Sus scrofa*), fallow deer (*Dama dama*) and roe deer (*Capreolus capreolus*) (Broekhuizen et al., 2016). Hunting levels in the Veluwe area can be up to 9.48 ungulates km^−2^ year^−1^ (Ramirez et al., 2021).

### 2.2 Data collection and preparation

**FIGURE 3:**
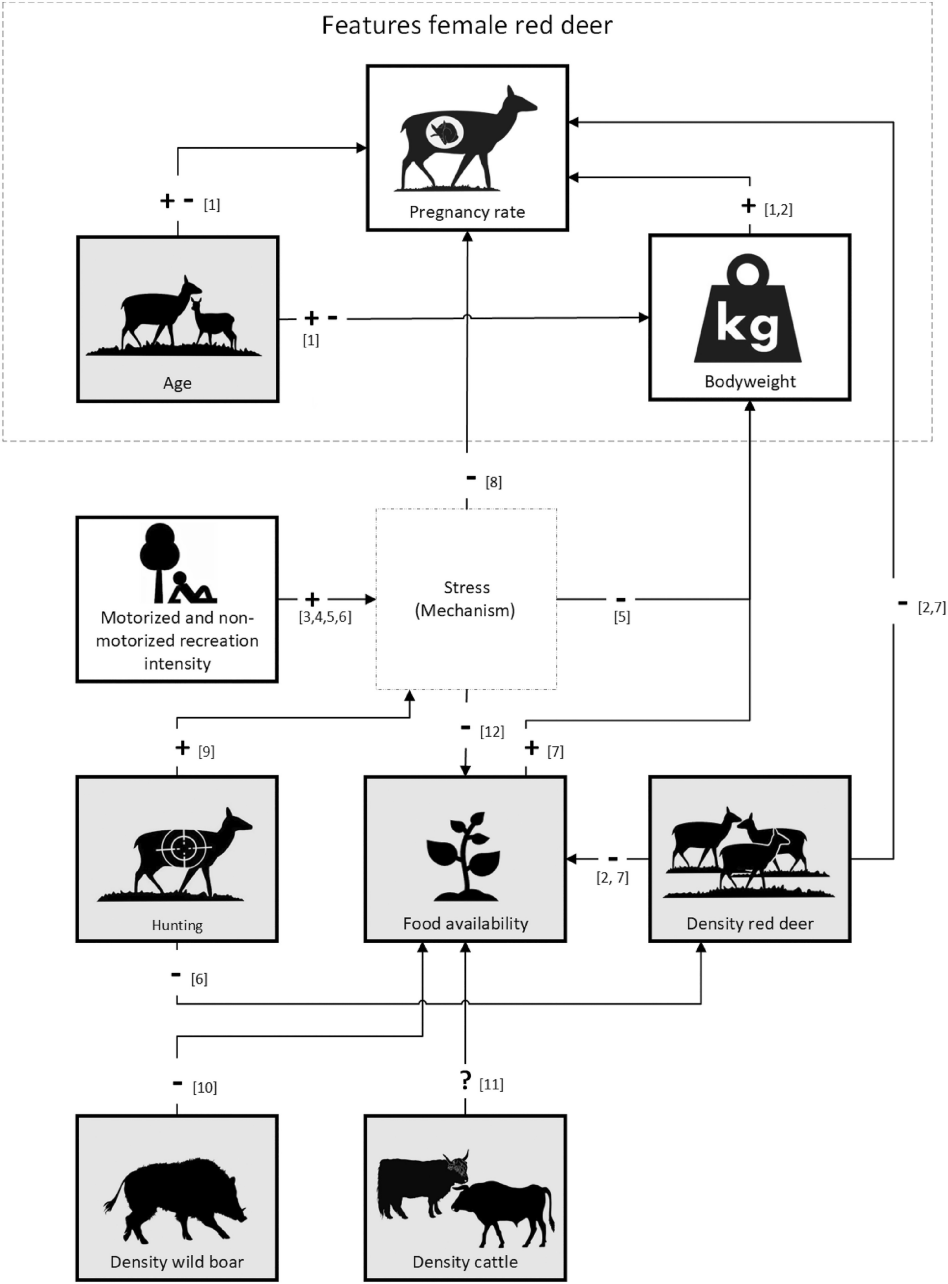
Conceptual representation of the effects of motorized and non‐motorized recreation intensity on female red deer bodyweight and pregnancy rates including control variables. Focus variables are indicated in white and control variables are indicated in gray. Non‐motorized recreation intensity includes dogs, cyclists, hikers and horse riders. Motorized recreational intensity includes cars and quads. + = positive effect; − = negative effect; ? = effect is unclear; +− = effect could be both positive and negative. (1) Borowik et al. (2016); (2) Putman et al. (2019); (3) Papouchis et al. (2001); (4) Larson et al. (2016); (5) Carpio et al. (2021); (6) Rodriguez‐Hidalgo et al. (2010); (7) Bötsch et al. (2020) (8) Vilela et al. (2020); (9) Barrios‐Garcia and Ballari (2012); (10) Kuiters et al. (2005); (11) Jayakody et al. (2008), Stankowich (2008).

We performed a quantitative study using a correlational research design to test our hypotheses. In order to do this, we collated data on recreational activities and red deer bodyweight and fecundity between 1985 and 2015 (see conceptual representation in Figure 3). We quantified fecundity as a pregnancy rate.

#### 2.2.1 Recreational activities

Yearly, between 1985 and 2015, the relative intensity of recreational activities was recorded by a single observer (R. Bijlsma) with the aim of assessing their effect on ground‐breeding bird ecology (see Bijlsma, 2006). The recreational activities were visually observed in one open landscape area (30 ha) split in two zones: Otterlose zand (open for public; 15 ha) and Mosselse zand (closed for public; 15 ha). Observations were done for a mean time (±SD) of 17.2 (±9.3) h (range: 6–45 h) spread across an average (±SD) of 8.0 (±3.9) days per year (range: 3–21 days) (Figure 2). For the majority of the time (86%), observations were done between March and August (i.e., peak of recreational activity in the Veluwe) (Bijlsma, 2006). We assumed that the recreational activities observed in the two zones were representative of the Veluwe area, especially to assess whether the relative recreation intensity (hereafter recreation intensity) significantly differed between various years. Recreationists were counted (with complete visual coverage) whilst walking without a fixed route but covering the whole area. Observations were divided into six different categories, namely: hikers, horse riders, vehicles (cars and quads), dogs (off‐leash) and cyclists (including All Terrain Bikes, ATBs). Using this data, we calculated the annual number of recreationists for each type observed per hour.

#### 2.2.2 Red deer characteristics

During the regular hunting season between 1985 and 2015, hunters culled an average of 82.3 (SD = 31.3) female red deer per year (total *n* = 488) between October and June in assigned locations within the area of the Game Management Unit (GMU) (Figure 2). The GMUs predetermine the number of animals to cull in each age class before the hunt to reduce population numbers and promote tree regeneration. Age and bodyweight are known to affect fecundity (Borowik et al., 2016). During that period, data on age and bodyweight were collected by Han ten Seldam and Dirk Lieftink for no specific purpose but to serve as a baseline for red deer demographic data. Age was defined through a post‐mortem examination. For red deer younger than 2.5 years, age was determined through incisor and molar changes. The age of older individuals was estimated from the wear of the teeth in the lower jaw (Lowe, 1967). After evisceration, female red deer were weighed (*n* = 261) and the presence or absence of a foetus was recorded.

#### 2.2.3 Characteristics of the study area

Because of our correlative study design, we collated data on population variables to control for their effects on bodyweight and fecundity (i.e., confounding effects). Red deer bodyweight and pregnancy rates can be influenced by the density of red deer (Carpio et al., 2021; Putman et al., 2019) and the density of competitor ungulates (Barrios‐Garcia & Ballari, 2012; Borowik et al., 2016). We did not control for roe deer density, as roe deer seem to be more affected by the presence of red deer than vice versa, possibly because roe deer are more selective in their foraging compared to red deer (Borkowski et al., 2021). Similarly, we did not control for fallow deer density, as there seems to be little competition between the sympatric fallow deer and red deer (Bartos et al., 2002). Therefore, we collated data on red deer density (*t*_−3_, *t*_−2_, *t*_−1_ and *t*_0_) and wild boar density estimated by hunters and game managers (Hušek et al., 2021), using a yearly census according to a fixed protocol (see Appendix S1) (Game Management Unit Gelderland, 2019). The census of red deer took place in March and April at count sites (1 per 400 ha) within fixed subareas (Figure 2), when animals got active and vegetation cover did not limit visibility. Wild boar were counted between May and June at fixed locations (1 per 200 ha) within the area of the GMU, using bait. Furthermore, we collated data on the densities of semi‐domesticated herds of Sayaguesa and Scottish Highland cattle that were present and monitored in the area since 2002. Moreover, we collated culling data of red deer from the Game Management Unit (GMU), to account for their effect on chronic stress (Vilela et al., 2020) and red deer density (Carpio et al., 2021; Stewart et al., 2005), and therefore bodyweight and fecundity (Bötsch et al., 2020; Putman et al., 2019).

Additionally, we collated environmental variables that are known to affect bodyweight and fecundity via food availability (Borowik et al., 2016), such as habitat availability, the presence of supplementary feeding sites and mean annual temperature and precipitation (Rodriguez‐Hidalgo et al., 2010). We assessed changes in habitat availability (Stankowich, 2008) using ArcMap (10.7.1) and LGN1–LGN7 Landsat images (WUR‐Alterra, 1980–2012). We quantified the available habitat for red deer by including habitat types related to foraging (i.e., grasslands, deciduous forest, coniferous forest, mixed forest and heath; Gebert & Verheyden‐Tixier, 2001) (Appendix S1: Figure S1). In addition, we recorded the presence and absence of supplementary feeding sites in the area. Nevertheless, even though chronic stress can reduce the intake of food (Zanette et al., 2013) and therefore affect the bodyweight and fecundity of animals, our study design did not allow us to take this into account. Moreover, we collected mean annual temperatures and precipitation from the nearest weather station (i.e., Deelen at 8.4 km from the study site), as it can affect energy expenditure, bodyweight and the production of biomass (i.e., food) (Rodriguez‐Hidalgo et al., 2010).

### 2.3 Data analysis

Data had been explored in SPSS (IBM SPSS statistics 28) following the protocol by Zuur et al. (2010). Because the different types of recreation were strongly correlated, we used a Principal Component Analysis with a varimax rotation to extract two components (96.9% of the total variance) with an eigenvalue larger than 1. We characterized the first component as a ‘non‐motorized’ axis due to its strong correlation with the number of dogs, hikers, cyclists (including ATBs) and horse riders (Table 1). The second component was characterized as a ‘motorized’ axis based on its strong correlation with the number of vehicles (including quads). Since the vehicle data contained a lot of zeros (60.9%), we transformed it to a presence‐absence variable.

**TABLE 1:** Principal component scores and eigenvalues of five types of recreational activities in the Veluwe nature area.

Type of recreational activity	Principal component 1	Principal component 2
Dog walking	**0.97**	0.01
Hiking	**0.95**	−0.01
Cycling	**0.90**	0.27
Horse riding	**0.78**	0.37
Vehicle driving	0.1	**0.98**
Eigenvalues (%)	3.44 (68.8)	1.00 (20.01)

*Note*: The main structuring variables relative to each axis are indicated in bold.

We took into account the following control variables: age, available habitat, presence of feeding sites, red deer density (*t*_−3_, *t*_−2_, *t*_−1_ and *t*_0_), wild boar density, cattle density, mean annual temperature and precipitation and the annual number of red deer culled (i.e., shot). The density of red deer and the densities of wild boar and cattle were square‐root transformed to reduce skewness. The continuous independent variables were standardized and assessed for multicollinearity (*r* > .7; Dormann et al., 2013). The correlation between categorical and continuous variables was visually assessed through overlap in boxplots. Multicollinearity was assumed in the absence of overlap, and therefore one of the variables examined was not included in the initial model. The initial model did not include wild boar density and cattle density because of their strong correlation with the non‐motorized variable. The annual number of culled red deer and red deer density (*t*_−1_) were excluded because of their strong correlation with red deer density (*t*_0_). Red deer density (*t*_−2_) was omitted due to its strong correlation with red deer density (*t*_−3_). Lastly, the mean annual temperature in years with feeding sites present was significantly lower than when absent, suggesting a spurious correlation, leading to the exclusion of the feeding sites variable from the initial model (see Appendix S1: Figure S2). After selection, the following control variables remained: age, density red deer (*t*_−3_ and *t*_0_), available habitat, mean annual precipitation and mean annual temperature.

We constructed two Generalized Linear Mixed Models (GLMMs) in R (v.4.2.1.; R Core Team, 2021) to assess the direct and indirect effect via bodyweight of two recreational components, motorized and non‐motorized, on red deer pregnancy rates. For the direct effect of recreation on pregnancy rates, we used a Binomial GLMM with a log‐link function with the glmmTMB package (v.1.1.4; Brooks et al., 2017). In addition to the control variables mentioned above, bodyweight was included as an independent variable. Age was square‐root transformed to reduce skewness. To obtain odds ratios and 95% Confidence Intervals, the model parameters function (parameters package, version 0.18.2; Lüdecke et al., 2020) was used. The inclusion of the random factor year in the pregnancy model, aimed at addressing the collection of data from multiple deer within a single year, proved to have little added value as the variance of this random factor was close to zero. To assess the effect of recreation on bodyweight, we used a Gaussian GLMM, which included the linear and quadratic terms of age (Putman et al., 2019) in addition to the control variables mentioned above and the random factor year.

We used the ‘drop1’ protocol of Zuur et al. (2009) to select the models with the lowest value for the Akaike Information Criterion with a correction for small sample sizes (Table 3). We then assessed the fit of both final models using residual diagnostics (see Appendix S1: Figures S3 and S4) and tested the pregnancy model for overdispersion and zero inflation using the DHARMa package (v.0.4.6; Hartig & Lohse, 2022). Finally, we used piecewise Structural Equation Modelling in R (Lefcheck, 2016) to perform a pathway analysis. To integrate the final models for pregnancy rates and body weight into a unified Structural Equation Model (SEM) (as illustrated in the conceptual representation in Figure 3), we utilized the psem function from the ‘piecewiseSEM’ package in R (Lefcheck, 2016). This function enabled the calculation of standardized path coefficients, providing a measure of the strength and direction of relationships between variables within the SEM. By standardizing the path coefficients, we obtained a consistent measure of the magnitude of the effects, enabling meaningful comparisons across different variables. Our analysis focused on assessing the direct effects of recreation intensity on body weight and pregnancy rates. Additionally, we explored the indirect and total effects of recreation intensity on pregnancy rates, considering body weight as a mediator.

## 3 RESULTS

**FIGURE 4:**
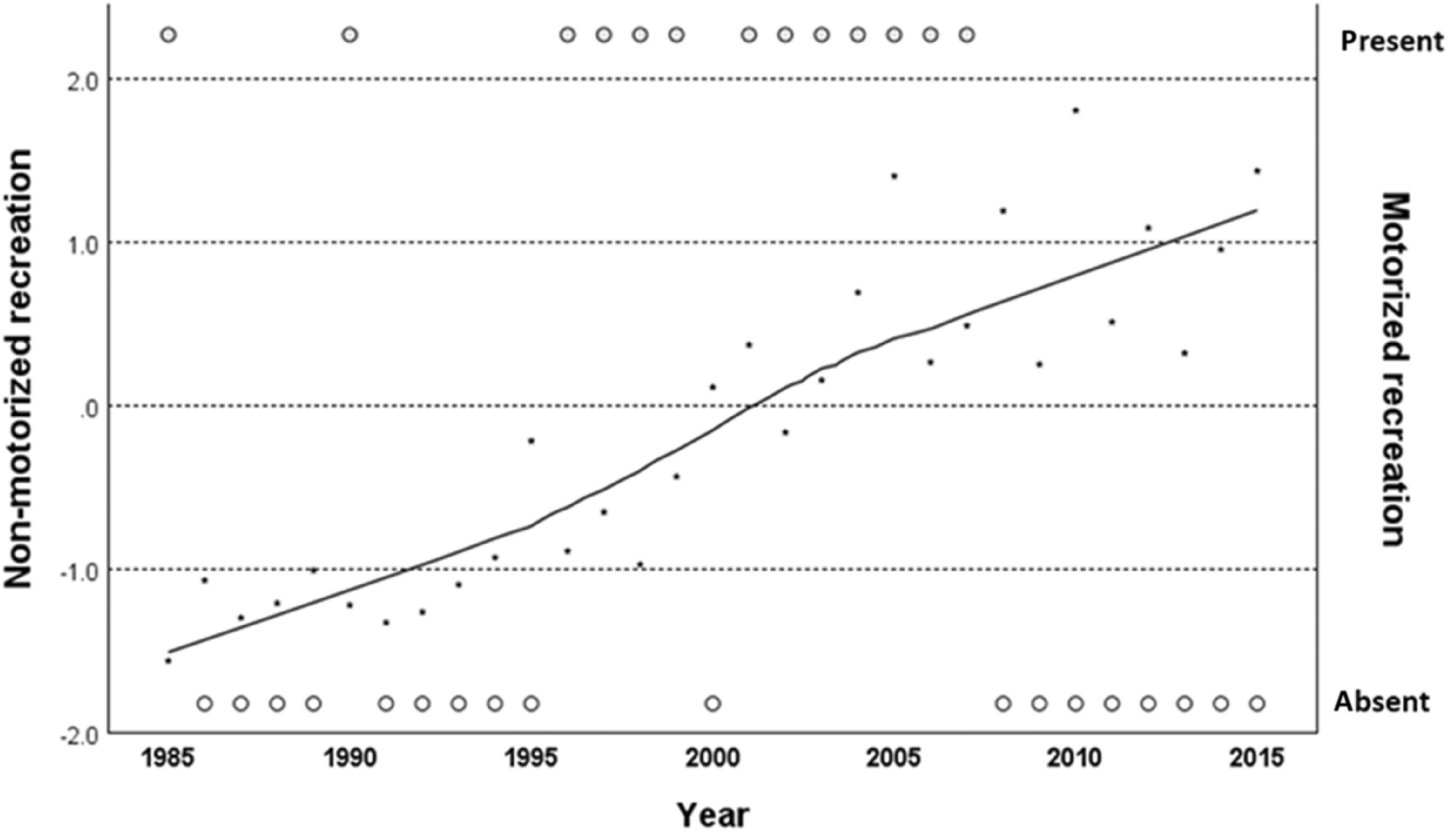
Standardized non‐motorized recreational intensity (*Z* score) and the presence and absence of motorized recreation in time (year) in the Veluwe national park between 1985 and 2015. Based on data provided by R. Bijlsma.

In total, 228 red deer out of 261 were pregnant (mean = 79.3 (±26.5%) pregnant per year) with an average bodyweight of 61 kg (SD = 8; range: 41–83 kg) (Table 2). The relative density of red deer in the area was on average 2.4 deer km^−2^ (SD = 0.4; range: 1.8–3.5). Between 1985 and 2015, the red deer pregnancy rate decreased by 10.2%, while the average female bodyweight decreased by 8 kg. The recreational intensity of non‐motorized activities strongly increased in time, while in general, motorized recreation was mainly present between 1996 and 2007 (Table 2, Figure 4).

**TABLE 2:** Characteristics (mean ± SD [range]) of recreational activities, red deer and the study area in three periods of 10 years.

	1985–1994	1995–2004	2005–2015
Recreational activities			
Non‐motorized recreation^a^	898 ± 310.4 (487–1500)	3619 ± 1476.6 (2451–6039)	6890 ± 1988.2 (4526–10,950)
Motorized recreation^b^	10 ± 20.7 (0–56)	87 ± 83.2 (0–244)	24 ± 42.8 (0–104)
Red deer (*n* per year)^c^	*n* = 6–22	*n* = 2–15	*n* = 1–6
Age (year)	4 ± 3.4 (1–12)	5 ± 4.9 (1–16)	2 ± 1.6 (1–6)
Bodyweight (kg)	63 ± 4.3 (56–70)	62 ± 6 (55–72)	55 ± 6 (50–67)
Red deer pregnancy rate (%)	91 ± 11.1 (67–100)	81 ± 15.1 (50–100)	67 ± 38.8 (67–100)
Study area			
Number of red deer shot (*n* per year)	93 ± 24.1 (62.5–122)	74 ± 27 (38–123)	93 ± 41.2 (31–145)
Number of hunting days (*n* per year)	70 ± 7.4 (62–77)^d^	75 ± 28.4 (39–133)	94 ± 36.7 (37–149)
Density red deer (red deer km^−2^)	3 ± 0.2 (2–3.7)	2 ± 0.3 (1.8–2.9)	3 ± 0.4 (2–3.5)
Available habitat (km^2^)	68 ± 1.6 (66.7–69.8)	66 ± 0.2 (65.8–66.2)	65 ± 2.9 (62–68.1)

^a^ Total number of dogs hikers cyclists (including ATBs) and horse riders.

^b^ Total number of vehicles (including quads).

^c^ Range of the number of red deer per year taken into account in our study in each 10 ‐year period.

^d^ In the period 1985–1994, the number of hunting days per year was only available from 1991 to 1994.

**TABLE 3:** Model selection red deer pregnancy rate and bodyweight model.

	AICc	ΔAICc	*w*
Pregnancy rate model			
PR ~ SQRT(A) + BW + MR + NMR	173.83	0.0	0.41
PR ~ SQRT(A) + BW + MR + NMR + HA	174.24	0.4	0.33
PR ~ SQRT(A) + BW + MR + NMR + HA + P	175.61	1.8	0.17
PR ~ SQRT(A) + BW + MR + NMR + HA + P + *T*	177.65	3.8	0.06
PR ~ SQRT(A) + BW + MR + NMR + HA + P + *T* + SQRT(D_0_)	179.72	5.9	0.02
PR ~ SQRT(A) + BW + MR + NMR + HA + P + *T* + SQRT(D_0_) + SQRT(D_−3_)	181.87	8.0	0.01
Bodyweight model			
BW ~ A + A^2^ + MR + NMR + P + (1|Year)	1731.42	0.0	0.30
BW ~ A + A^2^ + MR + NMR + P + HA + (1|Year)	1731.53	0.1	0.28
BW ~ A + A^2^ + MR + NMR + P + HA + SQRT(D_−3_) + (1|Year)	1732.01	0.6	0.22
BW ~ A + A^2^ + MR + NMR + P + HA + SQRT(D_−3_) + SQRT(D_0_) + (1|Year)	1732.99	1.6	0.14
BW ~ A + A^2^ + MR + NMR + P + HA + SQRT(D_−3_) + SQRT(D_0_) + *T* + (1|Year)	1734.92	3.5	0.05

Abbreviations: A, age; AICc, Akaike Information Criterion corrected for small sample sizes; BW, bodyweight; D_0_, density *t*_0_; D_−3_, density *t*_−3_; HA, habitat availability; MR, motorized recreation; NMR, non‐motorized recreation; P, mean annual precipitation; *T*, mean annual temperature; *w*, model weight.

**TABLE 4:** Summary of final model results with the effect of age bodyweight motorized recreation and non‐motorized recreation on pregnancy rates and bodyweight of female red deer (*n* = 261) between 1985 and 2015.

Pregnancy rate model^a^	*β* ^b^	SE	*p* Value	Odds ratio	95% CI
(Intercept)	2.08	0.34	<.001***		4.16, 15.53
sqrt(age)	−0.59	0.22	<.01**	0.55	0.36, 0.85
Bodyweight	1.24	0.29	<.001***	3.46	1.96, 6.10
Motorized recreation^c^	0.11	0.43	.80	1.11	0.48, 2.57
Non‐motorized recreation	−0.55	0.23	.02*	0.58	0.37, 0.90

Bodyweight model^d^	*β* ^b^	SE	*p* Value
(Intercept)	63.16	0.96	<.001***
Age	6.24	0.60	<.001***
Age^2^	−2.90	0.36	<.001***
Motorized recreation^c^	0.58	1.27	.65
Non‐motorized recreation	−1.69	0.72	.02*

*Note*: Control variables: density red deer (*t*_−3_ and *t*_0_), available habitat, mean annual precipitation and mean annual temperature were dropped during model selection and did not affect pregnancy rates and bodyweight.

^a^ Pregnancy rate ~ sqrt(age) + bodyweight + motorized recreation + non‐motorized recreation (the predictors of the pregnancy rate model cause a 24% reduction in the absolute value of the log‐likelihood parameter).

^b^ Beta's of continuous independent variables are standardized.

^c^ Reference value = presence.

^d^ Bodyweight ~ age + age^2^ + motorized recreation + non‐motorized recreation + (1|year) (*σ* ^2^ year: 5.27 (± 2.30) total variance explained: 30%).

*p < .05 **p < .01 ***p < .001.

**FIGURE 5:**
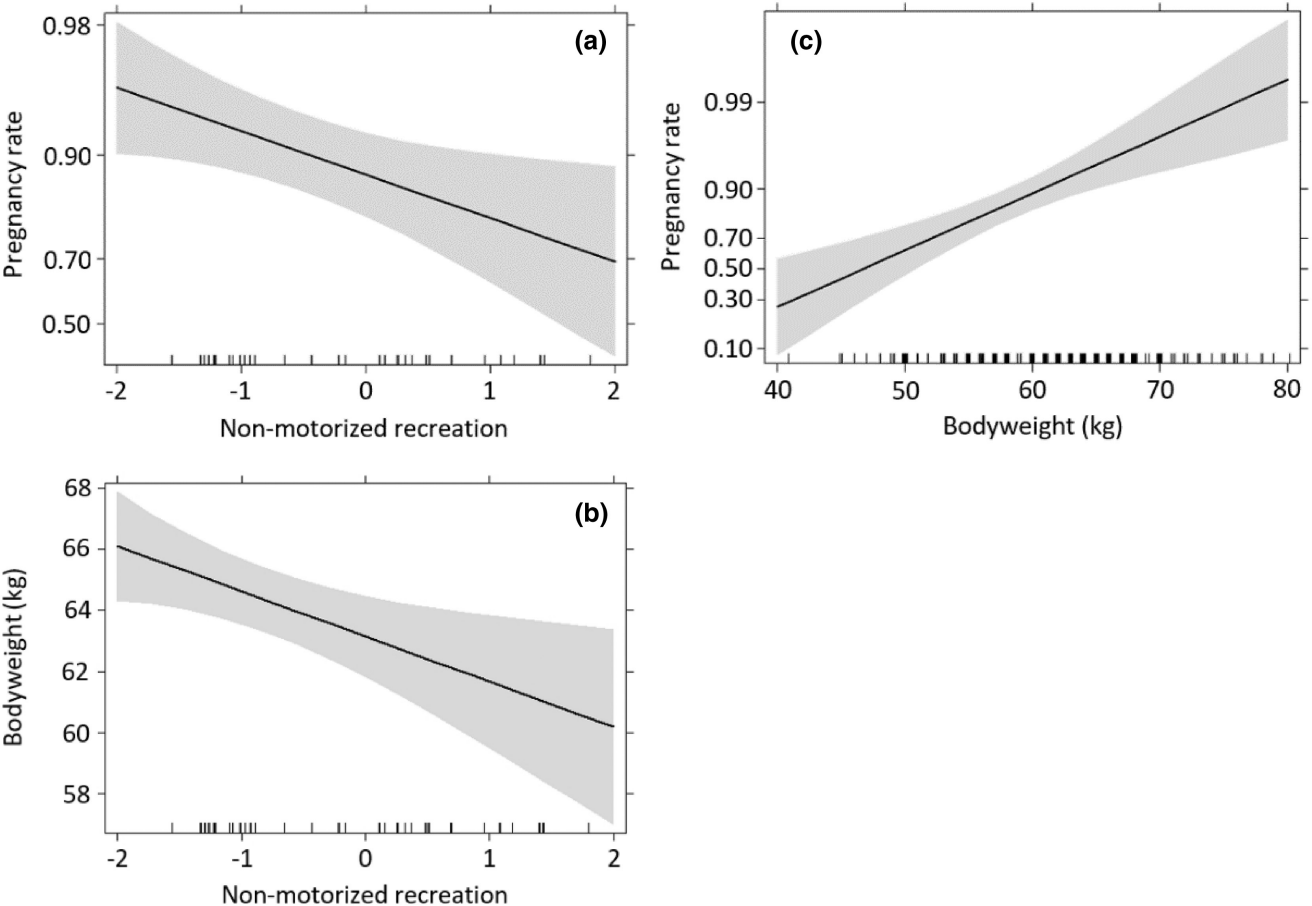
Effects of non‐motorized recreation on (a) pregnancy rates and (b) bodyweight and the effect of (c) bodyweight on pregnancy rates of female red deer (*n* = 261) between 1985 and 2015.The component non‐motorized recreation is extracted from a principal component analysis of dogs hikers cyclists (including ATBs) and horse riders.

In the final models, non‐motorized recreation had a strong direct negative effect on red deer pregnancy rates (Figure 5a, Table 4) (*β* = −0.59) and an even stronger negative effect on bodyweight (Figure 5b) (*β* = −1.69). Motorized recreation did not affect pregnancy rates and bodyweight. Bodyweight had the strongest relative (positive) effect on pregnancy rates (Figure 5c) (*β* = 1.24). Age was quadratically related to bodyweight. At an age of 7.26 years, female red deer attained their maximum bodyweight. Furthermore, the square root of age was linearly related to red deer fecundity, with fecundity decreasing with older females. Except age, none of the other control variables, density red deer (*t*_−3_ and *t*_0_), available habitat, mean annual precipitation and mean annual temperature, affected red deer pregnancy rates and body weight.

**FIGURE 6:**
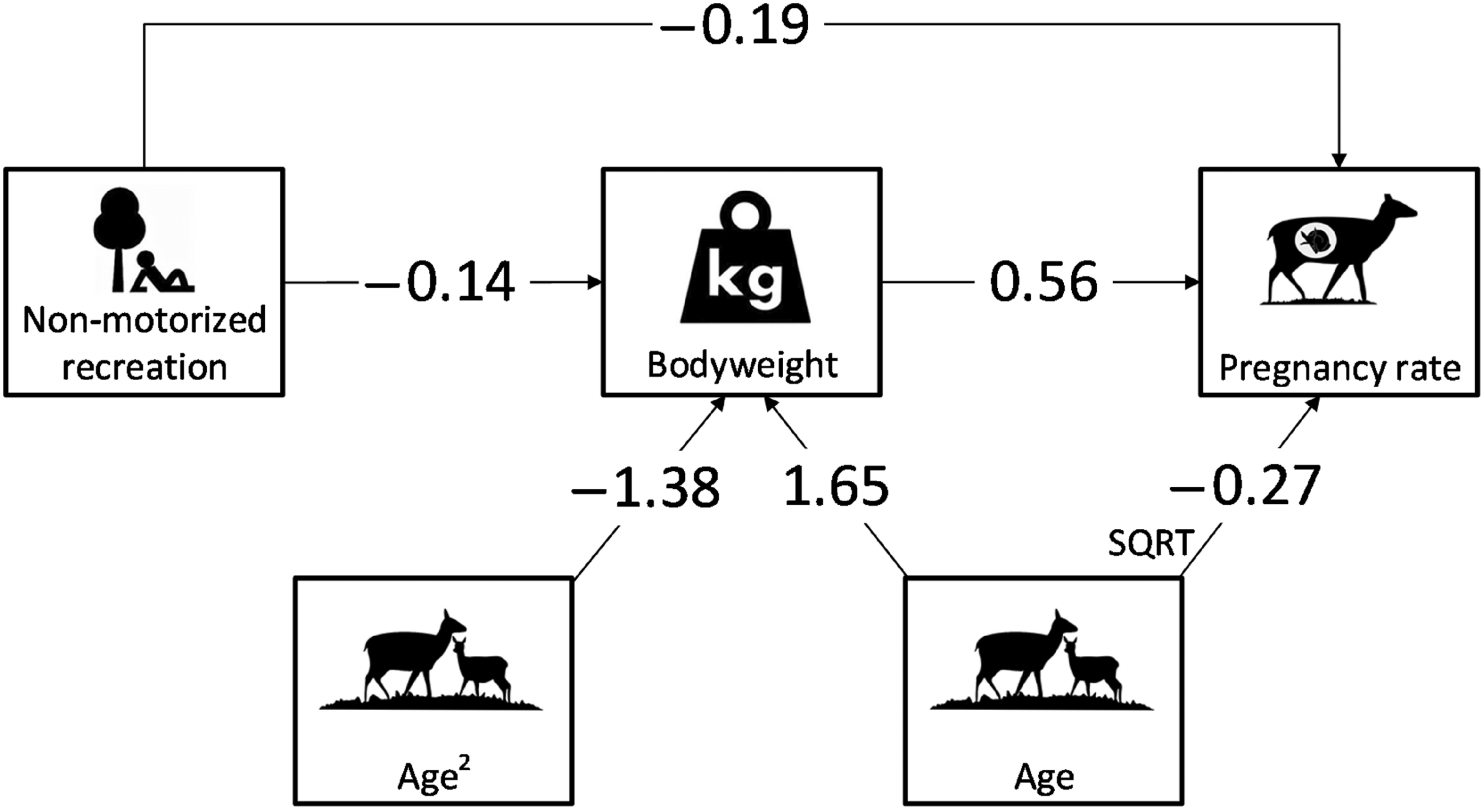
Final model structure and path coefficients (i.e. standardized regression coefficients) that show the direct effect of non‐motorized recreation on pregnancy rates and bodyweight and the direct effect of bodyweight on pregnancy rates. Effect size path coefficients (Cohen, 1992): large effect = 0.50 medium effect = 0.30 small effect = 0.10.

Based on a pathway analysis with standardized beta's (Figure 6), the indirect effect of non‐motorized recreation on pregnancy rates, with bodyweight as a mediator, was 29% of the total effect (−0.27). Therefore, the direct effect of non‐motorized recreation on red deer pregnancy rates (−0.19) is much larger than the indirect effect via bodyweight (−0.08).

## 4 DISCUSSION

Our study investigated the effect of fear of humans on the fitness of a large‐bodied ungulate over a 31‐year period. It is important to understand the fitness consequences of the fear of humans as an important stressor on other species for the management and conservation of these species (Darimont et al., 2015; Larson et al., 2016; Naidoo & Burton, 2020). In particular, it could enhance our understanding of the mechanisms that can result in trophic cascades (Chitwood et al., 2022), because large‐bodied ungulates can structure landscapes such as forests (Ramirez et al., 2018, 2023), affecting forest‐related plant and animal species (Svenning et al., 2015). Large‐bodied ungulates may be expected to fear humans more strongly than other predators, such as wolves, thereby exhibiting strong responses to humans (see Ciuti et al., 2012; Crawford et al., 2022; Zbyryt et al., 2018), especially hunters (Cromsigt et al., 2013). However, compared to hunting, species responses to recreation are generally considered less strong (but can vary in time and space) (Gundersen et al., 2021), in particular if recreation is restricted to trails (Reimers & Colman, 2006). Yet, Mols et al. (2021) showed that in contrast to recreation, hunting did not have an additive year‐round browsing impact on woody vegetation on a large scale, possibly because hunting is limited in time. Besides, repeated exposure to humans can also lead to habituation to fear (Blumstein, 2016). Nevertheless, in line with our first hypothesis, we found a direct negative correlation between the intensity of non‐motorized recreation and the pregnancy rates of red deer. This corroborates Putman and Langbein (2003) that found similar effects of human disturbance on deer fecundity in parks open to the public. Besides, recreation correlated negatively with bodyweight, whilst bodyweight was strongly positively related to the pregnancy rates of red deer (e.g. Borowik et al., 2016; Creel, 2018; Creel et al., 2014; Putman et al., 2019). This substantiates our second hypothesis that recreational activities can have an indirect effect on pregnancy rates with bodyweight as a mediator. Even though we assumed that repeated recreation could lead to chronic stress, ultimately affecting the pregnancy rate of red deer, we did not measure chronic stress. Boonstra (2013) suggested that chronic activation of the stress axis is an evolved adaptive response to variation in risk. Red deer possibly adapted to the recent increase (i.e., the last couple of decades) in disturbance by recreational activities, as adaptive evolution can operate on a generation‐to‐generation time scale (Bonnet et al., 2022). Nevertheless, both Boonstra (2013) and Creel (2018) argue that acute (unpredictable) stressors (e.g., a risky encounter) can have stress‐mediated costs, in particular if they impact an animal's stress physiology for a longer duration. Bijlsma (2006) has shown that visitors in the Veluwe area increasingly ventured into restricted areas between 1968 and 2005, which enhances the frequency of unpredictable encounters with wildlife. Besides, some activities affect wildlife more significantly than others because of their unpredictability (Stankowich, 2008). Additionally, first‐time visitors (10%–30% in the Veluwe) behave less predictable, as they more often do not use pedestrian trails and take longer walks away from the designated gateways compared to regular visitors (Beunen et al., 2008). It is therefore probable that frequent encounters with recreational activities could result in chronic stress in red deer in the Veluwe (see also Zbyryt et al., 2018).

Even though recreational activities can affect the fecundity of species (Bötsch et al., 2020; Larson et al., 2016), knowledge about the mechanisms that affect the responses of ungulates to human activities like recreation is far from complete. Besides, different recreational activities could have interactive or cumulative effects (Spaul & Heath, 2016). Recent studies suggest that the responses of ungulates to cues related to non‐lethal activities of recreationists could have consequences on the population level if ungulates incorrectly perceive these cues as risky (i.e., over‐response) (see assessment mismatch hypothesis; Smith, Gaynor, & Suraci, 2021). Habitat characteristics can modify or affect the risk assessment of ungulates (Weterings, Meister, et al., 2022). Especially in open habitats like the heathland areas in the Veluwe, animals have ample visibility and show increased perceptions of risk when disturbed compared to closed habitats (Stankowich, 2008). Next to this, population dynamics are suggested to be driven by a response mismatch (Smith, Gaynor, & Suraci, 2021) if ungulates display a generalized antipredator response to all cues related to non‐lethal activities initiated by humans. This could be the case when ungulates associate humans with the potential presence of a dog or hunting activities (Stankowich, 2008), which can both be associated with increased levels of risk (Cromsigt et al., 2013; Darimont et al., 2015).

Our results showed standardized effect sizes ranging from small to medium (Cohen, 1992), which corroborates Stankowich and Blumstein (2005). Possibly, these effect sizes are relatively high because of the presence of hunting in the Veluwe, which could exacerbate species' responses to recreation (Thiel et al., 2007), especially in areas with a high level of disturbance (Ciuti et al., 2012). Nevertheless, we did not investigate the interaction between recreation intensity and hunting on red deer body weight and pregnancy rates. Moreover, hunting in the Veluwe is done only within a limited time period during the year (Mols et al., 2021) with high‐powered rifles that are more difficult to associate with a predictable cue (Cromsigt et al., 2013; Ramirez et al., 2021). Besides, recreation in protected areas can be more substantial than recreation in areas that are not protected, because areas that are protected attract visitors (Reinius & Fredman, 2007). This is also seen happening in protected areas around the world where recreation has increased in great numbers (Vallecillo et al., 2019). As a result, recreation in protected areas with a high intensity of use can have a strong impact on species ecology (see, e.g. Gundersen et al., 2021; Schulze et al., 2018). Our results primarily emphasize the importance of regulating recreational activities in sensitive locations, such as protected areas. But, even though the Veluwe has zones restricted to the public (Province of Gelderland, 2022), recreational activities in restricted areas do take place and their intensity is strongly correlated to the recreational intensity in areas open to the public, however at a lower level (Bijlsma, 2006). Notably, visitors should be more actively informed by protected area managers about the effects of recreation on wild animal populations and their surroundings, as most visitors are unaware about the consequences of their activities (Gruas et al., 2020). In addition to animals, this could also benefit vegetation and soils, as recreation is an important source of pressure on the environment (Ballantyne & Pickering, 2013). This requires protected‐area management to be knowledge‐driven, evidence‐based, site‐specific and effective (Pressey et al., 2015), especially when related to the management of recreational pressure and its corresponding effects on nature values.

Corroborating our third hypothesis, motorized recreation showed less effect on red deer fecundity than non‐motorized recreation. On the one hand, this could apply to other species as well (Larson et al., 2016; Stankowich, 2008). For example, coyote (*Canis latrans*), bobcat (*Lynx rufus*) (George & Crooks, 2006) and wolverine (*Gulo gulo*) (Krebs et al., 2007) showed no responses to motorized recreation as opposed to non‐motorized recreation. On the other hand, non‐motorized recreation can affect species ecology in many ways (Larson et al., 2016). For example, bighorn sheep showed considerable individual heterogeneity in responses to human activities (Papouchis et al., 2001), while differences in the behaviour of recreationists (e.g., more threatening behaviour) can affect animal risk perception and result in larger flight distances (Stankowich, 2008).

Because we used a correlative study design, we could not ascertain causality between recreational intensity, body weight and fecundity, as such, we controlled for potential confounding factors (Ruxton & Colegrave, 2016). As expected, age was strongly correlated with red deer bodyweight and fecundity (Borowik et al., 2016), while these relationships were identical to the results of Putman et al. (2019). Besides age, we took various confounding variables into account to control for their effect on fecundity and bodyweight (see Tarlow & Blumstein, 2007), in particular mean annual temperature and precipitation, food supplementation, available habitat and population density. However, we did not find an effect of the mean annual temperature and precipitation. Possibly, this is because in different periods throughout the year, temperature and precipitation have a different effect on the quality and availability of food, red deer bodyweight and fecundity (Schmidt et al., 2001), which could be negated at a large temporal scale. Additionally, we did not find density‐dependent effects on fecundity and bodyweight. Possibly, the effects of density dependence on red deer reproduction and body condition operate only in resource restricted areas (Putman et al., 2019; Stewart et al., 2005), in contrast to our Veluwe area (Ramirez et al., 2021). Moreover, density dependence is not shown in populations at equilibrium (Clutton‐Brock et al., 1985). The relative densities of our population did not vary that much between 1985 and 2015 and fluctuated around 2.4 deer km^−2^, making it more difficult to detect density dependence. Similarly, the low variation in available habitat made the effects of this variable on fecundity and body weight difficult to detect. Nevertheless, diseases and parasites could possibly affect bodyweight and fecundity of red deer, but they were not taken into account in this study. Finally, we only sampled a single location to get a relative measure of recreation intensity between years. Possibly, data collection at this location did not represent the true development of recreation intensity in the Veluwe. However, the trend in recreation that we measured goes along with similar trends in other studies (see, e.g. De Boer & Langers, 2022; VisitVeluwe and Bureau of Economic Research Province of Gelderland, 2017). Therefore, our judgement is that the sample point to obtain a relative measure of recreation intensity over 31 years did not affect our results and conclusions.

In conclusion, recreational intensity was negatively correlated with the fecundity and body weight of red deer. Our results support the regulation of the number of recreationists in sensitive nature areas, especially non‐motorized recreationists such as hikers, horse riders and dog walkers.

## AUTHOR CONTRIBUTIONS

**Martijn J. A. Weterings:** Conceptualization (lead); data curation (lead); formal analysis (supporting); funding acquisition (lead); methodology (equal); project administration (lead); supervision (lead); writing – original draft (lead); writing – review and editing (lead). **Estella Y. C. Ebbinge:** Conceptualization (equal); formal analysis (equal); methodology (equal); software (equal); validation (equal); visualization (equal); writing – original draft (equal); writing – review and editing (equal). **Beau N. Strijker:** Conceptualization (equal); formal analysis (equal); methodology (equal); software (equal); validation (equal); visualization (equal); writing – original draft (equal); writing – review and editing (equal). **Gerrit‐Jan Spek:** Data curation (equal); resources (equal); supervision (supporting); writing – review and editing (equal). **Henry J. Kuipers:** Conceptualization (equal); formal analysis (lead); methodology (equal); supervision (equal); validation (lead); visualization (equal); writing – original draft (supporting); writing – review and editing (supporting).

## CONFLICT OF INTEREST STATEMENT

The authors declare that they have no conflict of interest.

## Supporting information


Appendix S1.

Appendix S2.

Data S1.

Data S2.

Data S3.

Data S4.

Data S5.

## Data Availability

The data base and R scripts are provided as supplementary data files, and are deposited in the Dryad Digital Repository [https://doi.org/10.5061/dryad.0rxwdbs7t].
