# Supplementary material for: Insights from a 31‐year study demonstrate an inverse correlation between recreational activities and red deer fecundity, with bodyweight as a mediator
Source: Ecol Evol. 2024 Apr 22;14(4):e11257. doi: 10.1002/ece3.11257 (PMC11035974; doi:10.1002/ece3.11257)
Supplement: Supplementary file 1 — Appendix S1. [file ECE3-14-e11257-s002.docx]

**Supplementary Materials**

I Protocol red deer and wild boar surveys

Red deer counts were performed twice-yearly (i.e., during dawn or dusk) using three days pre count, with one day in between. A single count took 3 hours (from 30 minutes before sunrise until 2.5 hours after, or from 2.5 hours before sunset until 30 minutes after). Every year, count sites within fixed locations (Fig. 2) were determined by coordinators of the Game Management Unit (GMU), in consultation with the keepers, based on the presence of red deer in the area. These sites were distributed across the area, with an average of one count site per 400 ha. Each count was preceded by instructing the participants and setting watches. Per site, two people were involved, a game warden or field expert of the GMU and an independent counter from a different GMU. Every team started and ended their counting simultaneously and used the same form to register the counts. Animals were counted through visual observations, using a binocular. Each team noted the number of animals in different age categories (stag, yearling-buck, hind, yearling-doe and fawn) and time and movements of the animals when counted. Afterwards, teams discussed their counts with adjacent teams to avoid double counts.

Wild boar were counted twice a year between May and June (with a maximum of seven days in between) at fixed locations (1 per 200 ha) within the GMU, using bait. These counts took place at fixed locations within the GMU area (Fig. 2) (1985 – 2015) with an average of one count site per 200 ha. Each count took three hours (from 2.5 hours before sunset until 30 minutes after). Bait was used to attract the wild boar to the counting sites and binoculars and infrared cameras were used to count the animals. The team composition and working method used for counting wild boar was identical to the afore-mentioned method for red deer. In the form used for counting wild boar, each team noted the date, start- and finishing time and the number of animals of different age and sex classes (i.e., boar, sow, pig of the sounder, piglet). If the group of animals left the count site, the direction and time they left was noted to avoid a double count by adjacent teams. Later, all data of the counts was registered by the coordinators and the total number of individuals per year was recorded in a database.

II Habitat availability red deer


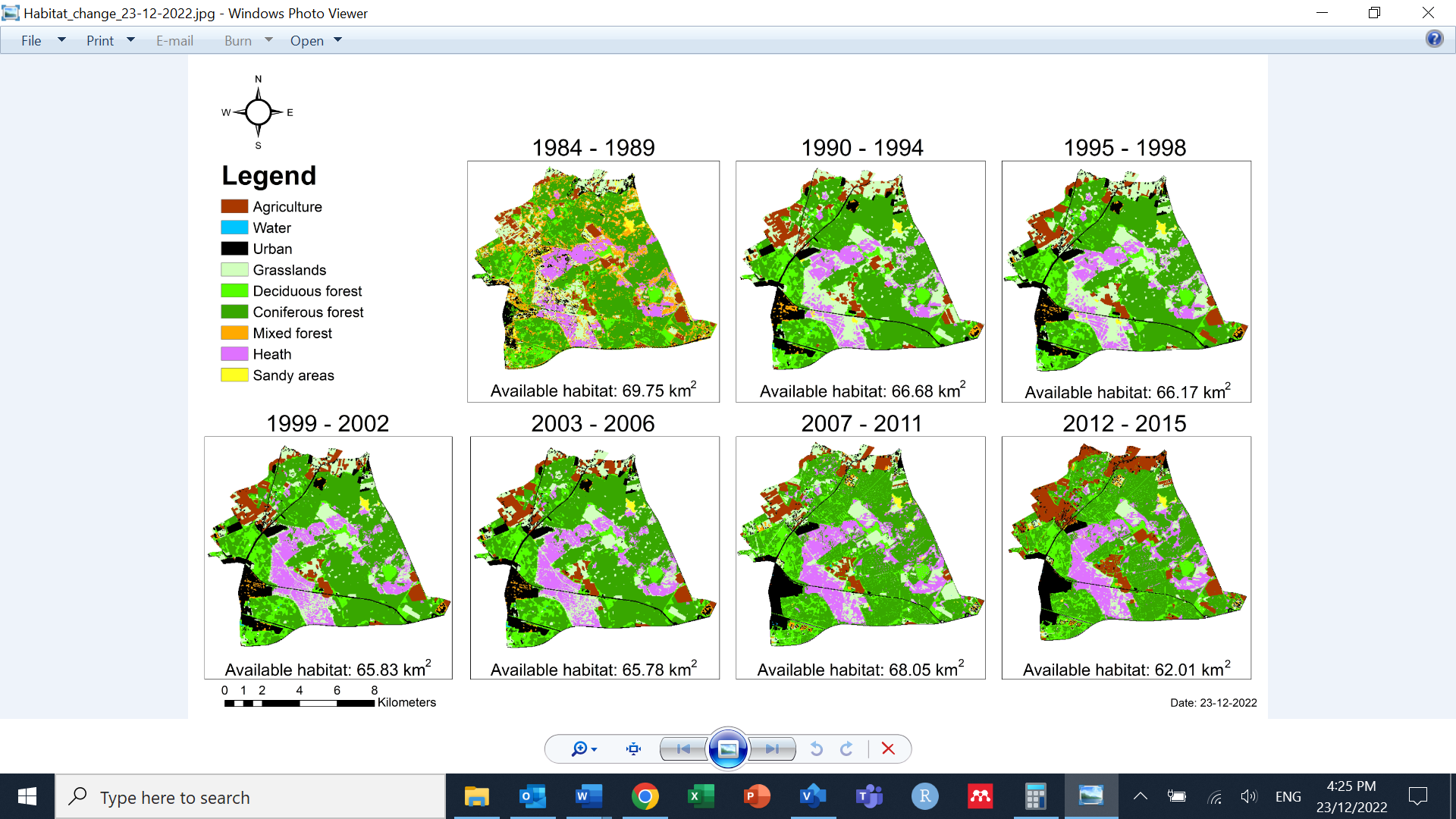

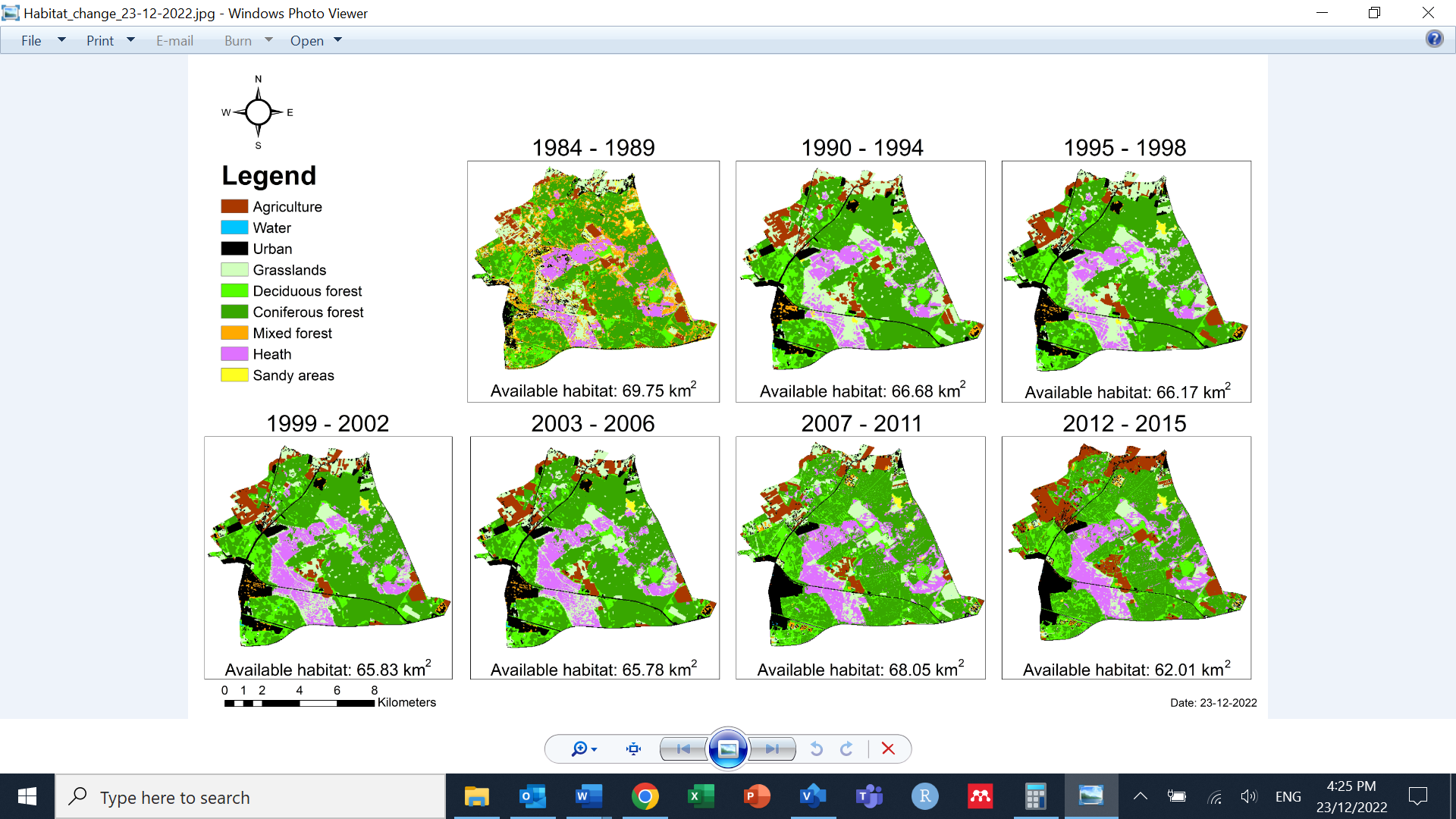


**Fig. S1** Available habitat for red deer in the Veluwe area between 1984 and 2015.

III Exploratory graphs feeding sites vs. mean annual temperature


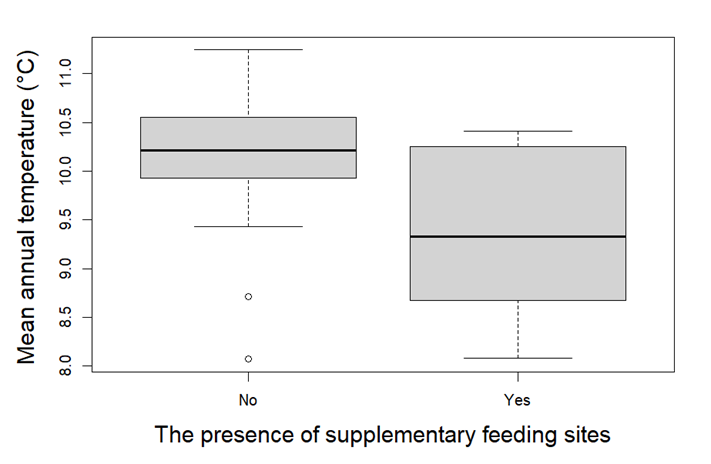


**Fig. S2** the difference between the mean annual temperature in years with feeding sites absent compared to years when feeding sites were present.

IV Residual diagnostics pregnancy rate model

# Final model pregnancy rate

ModPZ2a<- glm(Pregnantf ~ ZSQRT_Agen + ZWeightn + Vehiclef +

ZNmotor_recn, family = "binomial",

data = MydataAZ)

resPZ = simulateResiduals(ModPZ2a)

plot(resPZ)


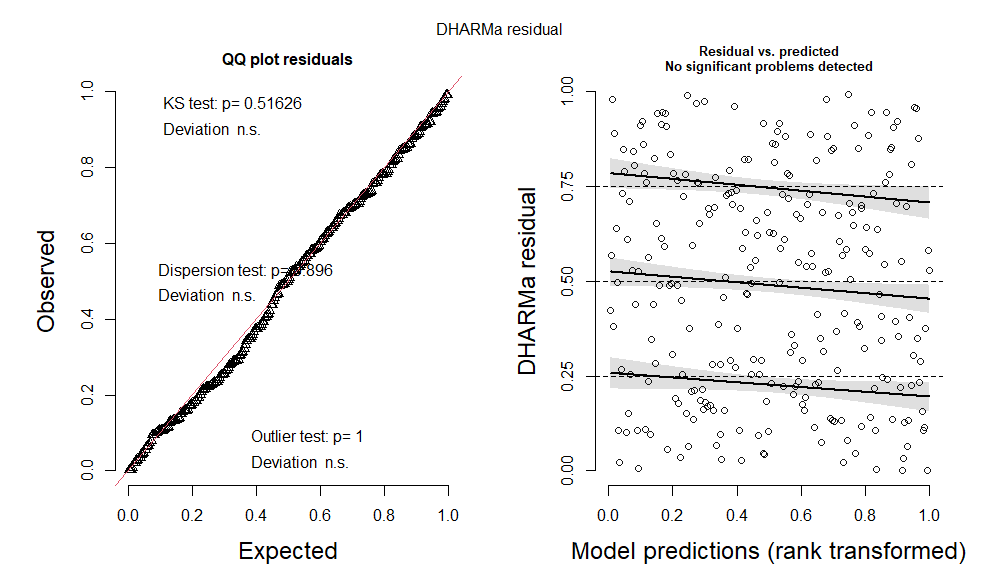


**Fig. S3** DHARMa standard residual plots of the pregnancy rate model.

DHARMa package R v.0.4.6; Hartig and Lohse (2022).

V Residual diagnostics bodyweight model

# Final model bodyweight

ModWZ2<- glmmTMB(Weightn ~ poly(ZAgen,degree=2,raw=TRUE) +

Vehiclef + ZNmotor_recn + (1 | Year),

REML = TRUE, dat = MydataAZ)

resWZ = simulateResiduals(ModWZ2)

plot(resWZ)


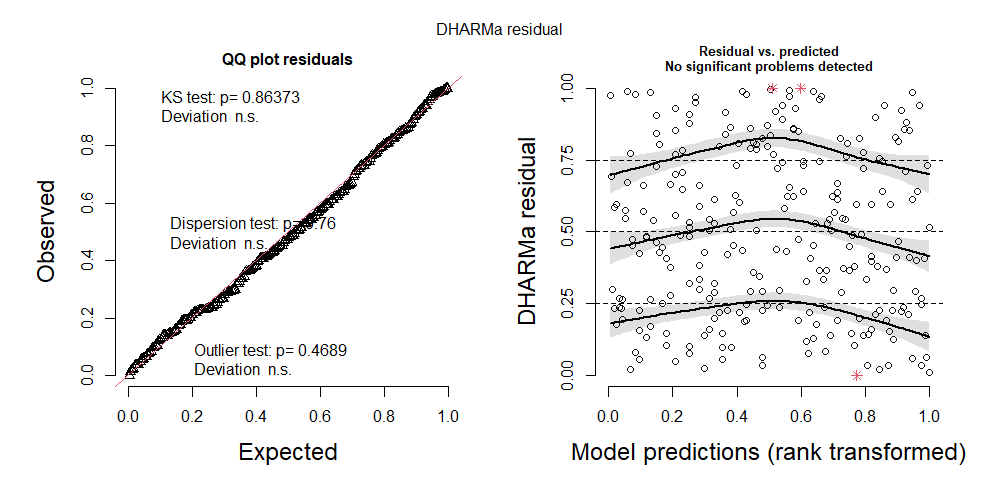


**Fig. S4** DHARMa standard residual plots of the bodyweight model.

DHARMa package R v.0.4.6; Hartig and Lohse (2022).
