# Supplementary material for: Insights from a 31‐year study demonstrate an inverse correlation between recreational activities and red deer fecundity, with bodyweight as a mediator
Source: Ecol Evol. 2024 Apr 22;14(4):e11257. doi: 10.1002/ece3.11257 (PMC11035974; doi:10.1002/ece3.11257)
Supplement: Supplementary file 2 — Appendix S2. [file ECE3-14-e11257-s006.docx]

This README file was generated on 2023-02-10 by Beau Strijker and Estella Ebbinge

GENERAL INFORMATION

1. Title of dataset: Data from: Insights from a 30-year study demonstrate an inverse correlation between recreational activities and red deer fecundity, with bodyweight as a mediator

2. Author Information

Corresponding Investigator

Name: Dr Martijn Weterings

Institution: Wageningen University and Research, Wageningen, Netherlands; Van Hall Institute, Leeuwarden, Netherlands

Email: martijn2.weterings@WUR.nl, martijn.weterings@HVHL.nl

Co-investigator 1

Name: Estella Ebbinge

Institution: Van Hall Institute, Leeuwarden, Netherlands

Co-investigator 2

Name: Beau Strijker

Institution: Van Hall Institute, Leeuwarden, Netherlands

Co-investigator 3

Name: Gerrit-Jan Spek

Institution: Vereniging Wildbeheer Veluwe/FBE Gelderland/Natuurlijk Fauna Advies Mts, Vaassen, Netherlands

Co-investigator 4

Name: Henry Kuipers

Institution: Van Hall Institute, Leeuwarden, Netherlands

3. Date of data collection: 1985-2015

4. Geographic location of data collection: Netherlands, Western Europe

5. Funding sources that supported the collection of the data: Nationaal Regieorgaan Praktijkgericht Onderzoek SIA (SVB/RAAK.PRO 318 02.048 to MW) and Van Hall Larenstein University of Applied Sciences

6. Recommended citation for this dataset: Weterings et al. (2023), Data from: Insights from a 30-year study demonstrate an inverse correlation between recreational activities and red deer fecundity, with bodyweight as a mediator, Dryad, Dataset

DATA & FILE OVERVIEW

1. Description of dataset

1a File list

File 1: Database_Annual

File 2: Database_Animal

1b Description

In database annual, all variables are presented per year (e.g. mean temperature). In database_animal, each row represents an individual red deer. Variables are presented on annual level, in the year the red deer was shot.

We collated data of recreational activities and red deer bodyweight and fecundity between 1985 and 2015. Recreational activities were observed by a single observer. Data on bodyweight and fecundity were obtained after red deer were shot. In addition, we collated data of control variables regarding red deer characteristics (i.e., age) and characteristics of the study area (i.e., available habitat, presence of feeding sites, red deer density (t-3, t-2, t-1 and t0), wild boar density, cattle density, mean annual temperature and precipitation and the annual number of red deer culled). We then constructed two Generalized Linear Mixed Models (GLMMs) in R (v.4.2.1.) to assess the direct and indirect effect via bodyweight of two recreational components, motorized and non-motorized, on red deer pregnancy rates. Finally, we used piecewise Structural Equation Modelling in R to perform a pathway analysis. We assessed the direct effects of recreation intensity on bodyweight and pregnancy rates, and the indirect and total effect of recreation intensity on pregnancy rates, with bodyweight as a mediator.

2. Data specific information

*refers to the name used in the R script, which might differ from the names used in the article

**Year** = the year in which the red deer was shot

**Pregnant** = presence (1) or absence (0) of a foetus
After evisceration the presence or absence of a foetus was recorded.

**Weight** = bodyweight in kg
After evisceration the red deer were weighed (n = 261).

**Age** = age in years
Age was defined through post-mortem examination of the shot red deer (n = 488). For red deer younger than 2.5 years, age was determined through incisor and molar changes. Age of older individuals was estimated from the wear of the teeth in the lower jaw.

*Data per year*

**Feeding sites** = presence (1) or absence (0) of supplementary feeding sites

**Red_deer** = total number of red deer

Red deer (*Cervus elaphus*) counts were performed twice (i.e., during dawn or dusk) in three days, with one day in between. A single count took 3 hours (from 30 minutes before sunrise until 2.5 hours after, or from 2.5 hours before sunset until 30 minutes after). Every year, count sites within fixed locations were determined by the coordinators of the GMU, in consultation with the hunting keepers, based on the occurrence of red deer in the area. These sites were divided over the area, with an average of one count site per 400 ha. Each count started at a central location, where all participants got instructions. Two people were counting per site, a game warden or field expert of the GMU and an independent counter from a different GMU. Every team started and ended their counting simultaneously and used the same form to register the counts. Animals were counted through visual observations, using a binocular. Each count team noted the number of animals in different categories (i.e., stag, yearling-buck, hind, yearling-doe and fawn) and the time and location of the animals when counted. Afterwards, all teams discussed their counts with adjacent teams to avoid double counts.

**D_RD** = Density of red deer, calculated from red deer counts (see ‘Red_deer’).

**Red_deer1,2,3** = total number of red deer in former years (t = 1 up to -3 years)

**D_RD1, D_RD2, D_RD3** = Density of red deer in former years (see ‘Red_deer1,2,3)

**WB** = total number of wild boar

Wild boar (*Sus scrofa*) were counted between May and June, at fixed locations (1 per 200 ha) within the GMU, using bait. twice each year, with a maximum of seven days in between. These counts took place at fixed locations within the GMU area (Figure 2) (1985 – 2015) with an average of one count site per 200 ha. Each count took three hours (from 2.5 hours before sunset until 30 minutes after). Bait was used to attract the wild boar to the counting sites and binoculars and infrared cameras were used to count the animals. The team composition and working method used for counting wild boar was identical to the afore-mentioned method for red deer. In the form used for counting wild boar, each team noted the date, start- and finishing time and the number of animals of different age and sex classes (i.e., boar, sow, pig of the sounder, piglet). If the group of animals left the count site, the direction and time they left was noted to avoid a double count by adjacent teams. Later, all data of the counts was registered by the coordinators and the total number of individuals per year was recorded in a database.

**D_WB** = Density wild boar, calculated from wild boar counts (see ‘WB’)

**Cattle** = total number of Sayagueas and Scottish Highland cattle

Semi-domesticated herds of Sayaguesa and Scottish Highland cattle were present and monitored in the area since 2002.

**D_C:** Density cattle, calculated from number of cattle (see ‘Cattle’)

**Vehicle_bin** = presence (1) or absence (0) of vehicles

Since the vehicle data contained a lot of zero’s, we transformed it to a binary variable.

**RD_shot** = total number of red deer shot

During the regular hunting season between 1985 until 2015, hunters harvested female red deer (n = 488) between October and June in assigned locations.

**Tr_col =** number of traffic collisions

**An_temp** = mean temperature in degrees Celsius.

We collected mean temperatures per day (in 0.1 degrees Celsius) from the nearest weather station (i.e., Deelen at 8.4 km 156 from the study site) to calculate the mean temperature per year in degrees Celsius.

**An_rain*** (precipitation) = mean precipitation in millimetres

We collected mean precipitation per day (in 0.1 millimetres) from the nearest weather station (i.e., Deelen at 8.4 km 156 from the study site) to calculate the mean precipitation per year in millimetres.

**Forage*** (available habitat) = available habitat in km^2^

We quantified the available habitat for red deer by including habitat types related to foraging (i.e., grasslands, deciduous forest, coniferous forest, mixed forest and heath.

**Nmotor_Recn:** Non-motorized recreation (i.e. dogs, hikers, cyclists (including ATBs) and horse riders)

Because the different types of recreation were strongly correlated, we used a Principal Component Analysis (PCA) with a varimax rotation to extract two components with an eigenvalue larger than 1. We characterized the first component (PC1) as a ‘non-motorized’ axis, based on its strong correlation with dogs, hikers, cyclists (including ATBs) and horse riders (Table 1). The second component (PC2) was characterized as a ‘motorized’ axis based on its strong correlation with the number of vehicles (including quads).
